# Supplementary material for: The 21-Gene Recurrence Score Assay and Prediction of Chemotherapy Benefit: A Propensity Score-Matched Analysis of the SEER Database
Source: Cancers (Basel). 2020 Jul 8;12(7):1829. doi: 10.3390/cancers12071829 (PMC7408834; doi:10.3390/cancers12071829)
Supplement: Supplementary file 1 [file cancers-12-01829-s001.pdf]

SupplementaryMaterials:

Table S1. Baseline characteristics according to chemotherapy in the primary unmatched cohort.

Characteristic	RS 0-10		*P*	RS 11-25		*P*	RS 26-100		*P*
	No chemotherapy (N = 18283)	Chemotherapy (N = 453)		No chemotherapy (N = 48904)	Chemotherapy (N = 8484)		No chemotherapy (N = 4855)	Chemotherapy (N = 8423)	
**Year**			< 0.001			<0.001			<0.001
2004-2006	697 (3.8%)	44 (9.7%)		2694 (5.5%)	752 (8.9%)		374 (7.7%)	532 (6.3%)	
2007-2009	3540 (19.4%)	167 (36.9%)		10901 (22.3%)	2674 (31.5%)		1228 (25.3%)	2114 (25.1%)	
2010-2012	5803 (31.7%)	135 (29.8%)		15865 (32.4%)	2666 (31.4%)		1624 (33.5%)	2660 (31.6%)	
2013-2015	8243 (45.1%)	107 (23.6%)		19444 (39.8%)	2392 (28.2%)		1629 (33.6%)	3117 (37.0%)	
Patient age, years (range)			<0.001			<0.001			<0.001
≤50	3770 (20.6%)	194 (42.8%)		12304 (25.2%)	3664 (43.2%)		860 (17.7%)	2345 (27.8%)	
>50	14513 (79.4%)	259 (57.2%)		36600 (74.8%)	4820 (56.8%)		3995 (82.3%)	6078 (72.2%)	
Race			0.135			<0.001			0.118
White	15169 (83.0%)	358 (79.0%)		40968 (83.8%)	6944 (81.8%)		3964 (81.6%)	6808 (80.8%)	
Black	1359 (7.4%)	43 (9.5%)		3558 (7.3%)	679 (8.0%)		460 (9.5%)	826 (9.8%)	
Others	1645 (9.0%)	50 (11.0%)		4096 (8.4%)	820 (9.7%)		398 (8.2%)	752 (8.9%)	
Unknown	110 (0.6%)	2 (0.4%)		282 (0.6%)	41 (0.5%)		33 (0.7%)	37 (0.4%)	
T category			<0.001			<0.001			< 0.001
T1	14537 (79.5%)	283 (62.5%)		39382 (80.5%)	6084 (71.7%)		3521 (72.5%)	5847 (69.4%)	
T2	3746 (20.5%)	170 (37.5%)		9522 (19.5%)	2400 (28.3%)		1334 (27.5%)	2576 (30.6%)	
Histologic type			0.657			<0.001			<0.001
IDC	13289 (72.7%)	333 (73.5%)		34780 (71.1%)	6217 (73.3%)		3940 (81.2%)	7081 (84.1%)	
IDC+ILC	1231 (6.7%)	33 (7.3%)		3959 (8.1%)	695 (8.2%)		294 (6.1%)	393 (4.7%)	
ILC	1612 (8.8%)	42 (9.3%)		6343 (13.0%)	983 (11.6%)		344 (7.1%)	437 (5.2%)	
Others	2151 (11.8%)	45 (9.9%)		3822 (7.8%)	589 (6.9%)		277 (5.7%)	512 (6.1%)	
Histologic grade			<0.001			<0.001			<0.001
1	6734 (36.8%)	129 (28.5%)		15577 (31.9%)	1744 (20.6%)		586 (12.1%)	548 (6.5%)	
2	9879 (54.0%)	243 (53.6%)		26844 (54.9%)	4744 (55.9%)		2300 (47.4%)	3506 (41.6%)	
3	1228 (6.7%)	66 (14.6%)		5274 (10.8%)	1794 (21.1%)		1873 (38.6%)	4247 (50.4%)	
Unknown	442 (2.4%)	15 (3.3%)		1209 (2.5%)	202 (2.4%)		96 (2.0%)	122 (1.4%)	
HR status			0.369			<0.001			0.645
ER+PR−	277 (1.5%)	4 (0.9%)		3906 (8.0%)	839 (9.9%)		1330 (27.4%)	2275 (27.0%)	
ER+PR+	18006 (98.5%)	449 (99.1%)		44998 (92.0%)	7645 (90.1%)		3525 (72.6%)	6148 (73.0%)	
Radiation therapy			0.378			<0.001			<0.001

No	7673 (42.0%)	200 (44.2%)	19299 (39.5%)	3714 (43.8%)	2655 (54.7%)	3585 (42.6%)
Yes	10610 (58.0%)	253 (55.8%)	29605 (60.5%)	4770 (56.2%)	2200 (45.3%)	4838 (57.4%)

Abbreviations: ER, estrogen receptor; HR, hormone receptor; IDC, invasive ductal carcinoma; ILC, invasive lobular carcinoma; PR, progesterone receptor; RS, recurrence score

Main text paragraph:

We noted an overall increase in the use of this assay over time (2004 through 2015; Table S1). Of these patients, 18,736 (21.0%) had RS <11, 57,388 (64.2%) had RS 11–25, and 13,278 (14.9%) had RS >25. The median age was 59 years (range:18–94 years); 25.9% were ≤50 years old. Most of the patients were white (83.0%). Approximately half (53.1%) had grade 2 tumors, 77.9% had tumors that were ≤2 cm in size, and 73.4% were diagnosed with invasive ductal carcinoma. There were more grade 3 tumors and fewer grade 1 tumors in the high RS group; this group also had fewer ER+PR+ tumors and more ER+PR− tumors than those were found among the lower RS groups. Chemotherapy use was reported as “yes” or “no/unknown”; chemotherapy use (“yes”) increased in proportion to 21-gene RS results with 2.4% (453/18,736) of those with RS <11, 14.8% (8484/57,388) of those with RS 11–25, and 63.4% (8423/13,278) of those with RS >25 receiving adjuvant chemotherapy. Clinical variables associated with a higher likelihood of receiving adjuvant chemotherapy were younger age at diagnosis (≤50 years), tumor size >2 cm, and high tumor grade (Table S1).

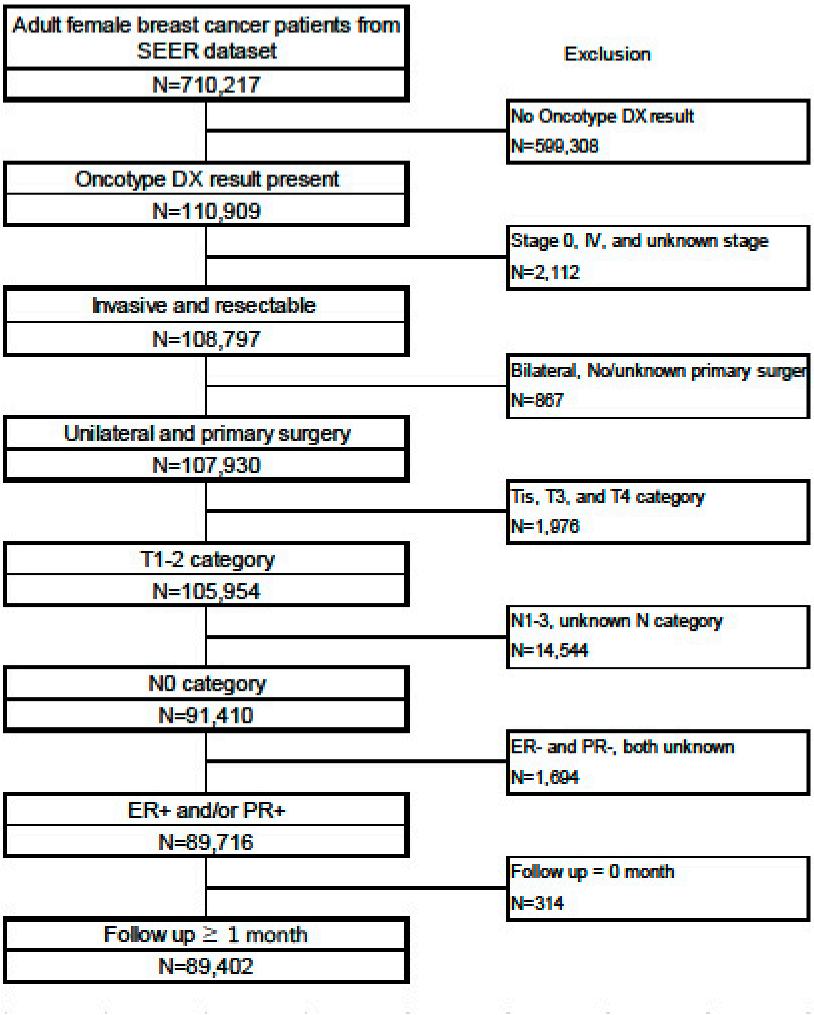

Figure S1. Study population and the process of case identification.

Main text paragraph:

The original primary cohort included a total of 89,402 patients with hormone receptor-positive, node-negative with T1 or T2 BC that had results from the 21-gene RS assay (Figure S1).

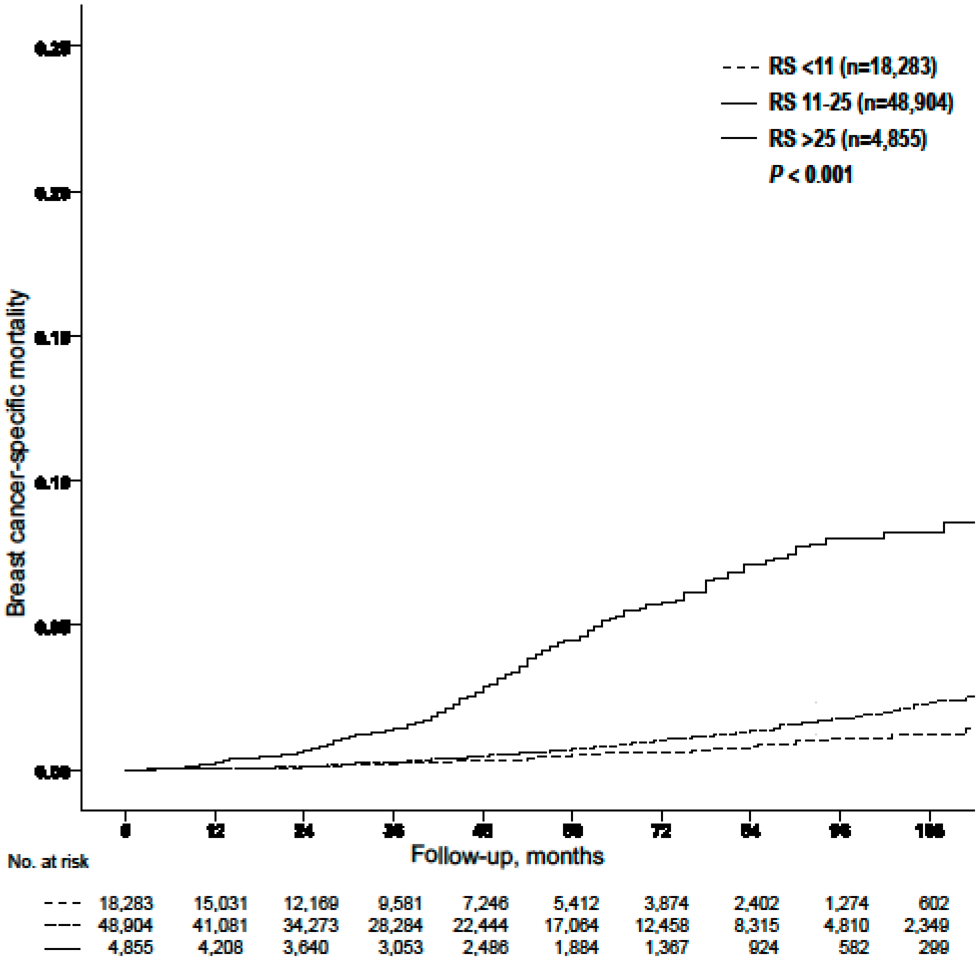

Figure S2. The Kaplan–Meier estimates of the risk of BC death by RS risk groups in the primary unmatched cohort. Chemotherapy-untreated patients with hormone receptor-positive, node-negative BC who had RS results of <11 (low), 11–25 (intermediate) and >25 (high) were included. Abbreviations: BC, breast cancer; RS, recurrence score.

Main text paragraph:

In the primary unmatched cohort, there were 18,283 patients with RS <11 who did not receive chemotherapy; for these patients, 5-year and 9-year BCSMs were 0.5% (95% CI, 0.3–0.7%) and 1.2% (95% CI, 0.8–1.6%). Also, for 48,904 patients with RS 11–25 who did not receive chemotherapy, 5-year and 9-year BCSMs were 0.7% (95% CI, 0.5–0.9%) and 2.4% (95% CI, 2.0–2.8%) (Figure S2).
